# Meta‐Analysis of the Influence of Integrated Traditional Chinese and Western Medicine on Cognitive Dysfunction After Hypertensive Intracerebral Hemorrhage

**DOI:** 10.1002/brb3.71083

**Published:** 2025-12-22

**Authors:** Yuexin Pan, Wenqian Pan

**Affiliations:** ^1^ Shandong University of Traditional Chinese Medicine Jinan China; ^2^ Department of Acupuncture Quanzhou Hospital of Traditional Chinese Medicine Quanzhou China

**Keywords:** cognitive dysfunction, hypertensive intracerebral hemorrhage, integrated traditional Chinese and Western medicine, meta‐analysis

## Abstract

**Background:**

Hypertensive intracerebral hemorrhage (HICH) constitutes a highly lethal form of cerebrovascular disease. A frequent consequence of HICH is cognitive dysfunction, which significantly impacts patients' quality of life. Despite the burgeoning interest in integrated traditional Chinese and Western medicine (ITCWM) for the rehabilitation of cognitive deficits following HICH, the available evidence supporting its efficacy remains limited.

**Aim:**

This study seeks to summarize the efficacy and safety of ITCWM in addressing cognitive dysfunction resulting from HICH using meta‐analysis.

**Methods:**

A literature search was conducted across the China National Knowledge Infrastructure, Wanfang, CQVIP, SinoMed, PubMed, Embase, Cochrane Library, and Web of Science databases. Studies were selected based on criteria. Quality assessment was evaluated through the Cochrane tool. Pooled results for continuous data were analyzed using the standardized mean difference (SMD) with a 95% confidence interval (CI). Binary categorical data were summarily analyzed using risk ratio (RR) and 95% CI.

**Results:**

A total of 13 RCTs involving 1123 patients were included. ITCWM significantly reduced NIHSS score (SMD = −2.36, 95% CI: −4.92, 0.19) and traditional Chinese medicine syndrome (TCMS) scores (SMD = −3.28, 95% CI: −4.02, −2.54), while improving activities of daily living (SMD = 3.67, 95% CI: 2.09, 5.26) and cognitive function scores (SMD = 1.27, 95% CI: 0.41, 2.14). The incidence of adverse events (RR = 0.26, 95% CI: 0.14, 0.46) was significantly lower in the ITCWM group. Subgroup analyses supported these findings, indicating that longer treatment durations (≥ 3 weeks) and multiple TCM therapies were associated with better outcomes.

**Conclusion:**

ITCWM demonstrates both effectiveness and safety in the management of post‐HICH cognitive dysfunction, improving neurological function, daily living abilities, and cognitive function while reducing adverse events.

## Graphical abstract

There is a growing interest in the methods of integrated traditional Chinese medicine and Western medicine (ITCWM) to treat cognitive dysfunction after hypertensive cerebral hemorrhage. In comparison with Western medicine treatment only, ITCWM could reduce NIHSS Score, improve ADL score, enhance cognitive function, reduce symptom burden, and reduce adverse events. Subgroup analysis showed that longer treatment (≥ 3 weeks) and multiple TCM therapies yield better outcomes. Sensitivity analysis indicated high stability for pooled results. In conclusion, ITCWM is an effective and safe therapeutic strategy for post‐HICH cognitive dysfunction, improving neurological recovery, daily living, and cognitive function.

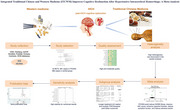

## 1 Introduction

Hypertensive intracerebral hemorrhage (HICH) represents a grave and often lethal and cerebrovascular condition, accounting for approximately 75% of all primary intracerebral hemorrhage cases in China (Jia et al. 2022). A prevalent sequela of HICH is cognitive dysfunction, which manifests in a significant number of cases (Shirzad et al. 2024; Y. Z. Zhang, Zhang et al. 2024). Research indicated that 30%–50% of HICH survivors experience varying degrees of cognitive impairment, which profoundly impacts their quality of life and social functioning (J. Zhang, Liu et al. 2024; Zuo et al. 2024). Despite the high incidence and severe ramifications associated with post‐HICH cognitive dysfunction, targeted treatments for post‐HICH cognitive dysfunction remain markedly scarce.

Integrated traditional Chinese and Western medicine (ITCWM) offers unique advantages in the management of post‐HICH cognitive dysfunction. Traditional Chinese medicine (TCM) therapies, including those aimed at promoting blood circulation to eliminate stasis and unblock orifices (including Tongqiao Huoxue decoction and Buyang Huanwu decoction), have demonstrated efficacy in facilitating hematoma absorption, minimizing brain edema, and enhancing neurological function. These beneficial effects are purported to stem from the modulation of the NLRP3 inflammasome and the inhibition of oxidative stress (Han et al. 2024; Wang, Yu et al. 2024; Yu et al. 2025). In addition, TCM interventions, such as acupuncture and herbal dietary formulations, have shown promise in improving daily living abilities of patients suffering from mild cognitive impairment (Wang, Lu et al. 2024; Xu et al. 2024). Modern Western medicine interventions, such as minimally invasive hematoma evacuation and postoperative cognitive training, significantly enhance motor and cognitive functions (Tan et al. 2024). However, the existing randomized controlled trials (RCTs) on ITCWM for post‐HICH cognitive dysfunction exhibit high methodological heterogeneity and insufficient sample sizes (W. Zhou, Liu et al. 2024).

In light of the limitations inherent in current treatments and the potential benefits of ITCWM, this study aimed to systematically assess the efficacy and safety of ITCWM in managing post‐HICH cognitive dysfunction using meta‐analysis. We posit that ITCWM will result in significant improvements in neurological deficits, daily living activities, and cognitive function, while simultaneously diminishing the incidence of adverse events in patients suffering from post‐HICH cognitive dysfunction. This investigation aims to provide a comprehensive assessment of ITCWM's therapeutic potential in this context, thereby yielding valuable insights for both clinical application and future research trajectories.

## 2 Materials and Methods

### 2.1 Literature Selection

All studies were meticulously selected from China National Knowledge Infrastructure, Wanfang, CQVIP, SinoMed, PubMed, Embase, Cochrane Library, and Web of Science databases, spanning from the establishment of the database through April 3, 2025.

The keywords employed for study selection included “hypertensive intracerebral hemorrhage,” or “cerebral hemorrhage,” and “cognitive impairment,” “cognitive dysfunction,” or “neurological deficit,” and “integrated traditional Chinese and Western medicine,” “Chinese herbal medicine,” or “acupuncture.”

### 2.2 Inclusion and Exclusion Criteria

The inclusion criteria contained (1) RCT, regardless of whether blinding was employed. (2) Participants were required to have confirmed diagnoses of HICH with cognitive impairment. (3) The age criterion set a minimum of 18 years, with no restrictions on gender or ethnicity. (4) The experimental group received ITCWM treatment, while the control group received only Western medicine treatment.

Exclusion criteria contained (1) not RCT studies (such as case reports, reviews, and animal experiments). (2) Incomplete data or inability to extract key results (such as only an abstract without full text). (3) Confounding intervention measures (such as combined with other alternative therapies or without Chinese medicine treatments). (4) Study subjects did not have HICH (such as traumatic or aneurysmal hemorrhage). (5) Study subjects were not patients with cognitive impairment.

### 2.3 Data Extraction

Data were meticulously compiled from all studies that met the predefined eligibility criteria. The extracted information included the name of the first author, publication year, number of cases, age range (expressed as mean ± standard deviation or median), and gender distribution (ratio of male to female) in the experimental group and control group. Furthermore, details regarding the interventions were gathered, comprising treatments involving Western medicine and TCM within the experimental group, treatment duration, and various outcomes (at least one of neurological deficit score, activity of daily living [ADL], and cognitive function score), total effective number, adverse events, and TCM syndrome integral scale (TCMS). The data were extracted by two researchers, respectively, and any differences were settled through collaborative discussion.

### 2.4 Evaluation of Literature Quality

The quality of the study was assessed using the Cochrane Tool implemented in RevMan 5.4. This assessment encompassed seven distinct dimensions, with each dimension categorized into three levels of risk: low risk, high risk, and uncertain risk.

### 2.5 Data Analysis

Pooled results were analyzed using RevMan 5.4. The *Q* test and the *I* ^2^ statistic test were used to detect heterogeneity of the included studies. If significant heterogeneity was identified (*p* < 0.1, or *I* ^2^ > 50%), the random effects model was adopted; otherwise, the fixed effects model was used. Pooled results of continuous variables were assessed via standardized mean difference (SMD) with 95% confidence interval (CI). Binary categorical variables were summarily evaluated by risk ratio (RR) and 95% CI. A subgroup analysis was performed based on therapy duration (comparing < 3 weeks vs. ≥ 3 weeks) and the type of TCM intervention (single TCM therapy vs. multiple TCM therapies), aiming to elucidate the sources of heterogeneity. Furthermore, sensitivity analysis and assessment of publication bias were conducted using Stata 12.0. Each individual study was systematically excluded to examine the robustness of the effect size and evaluate the sensitivity of the included literature. To assess publication bias, Begg's test was employed to generate funnel plots.

## 3 Results

### 3.1 Literature Selection

**FIGURE 1:**
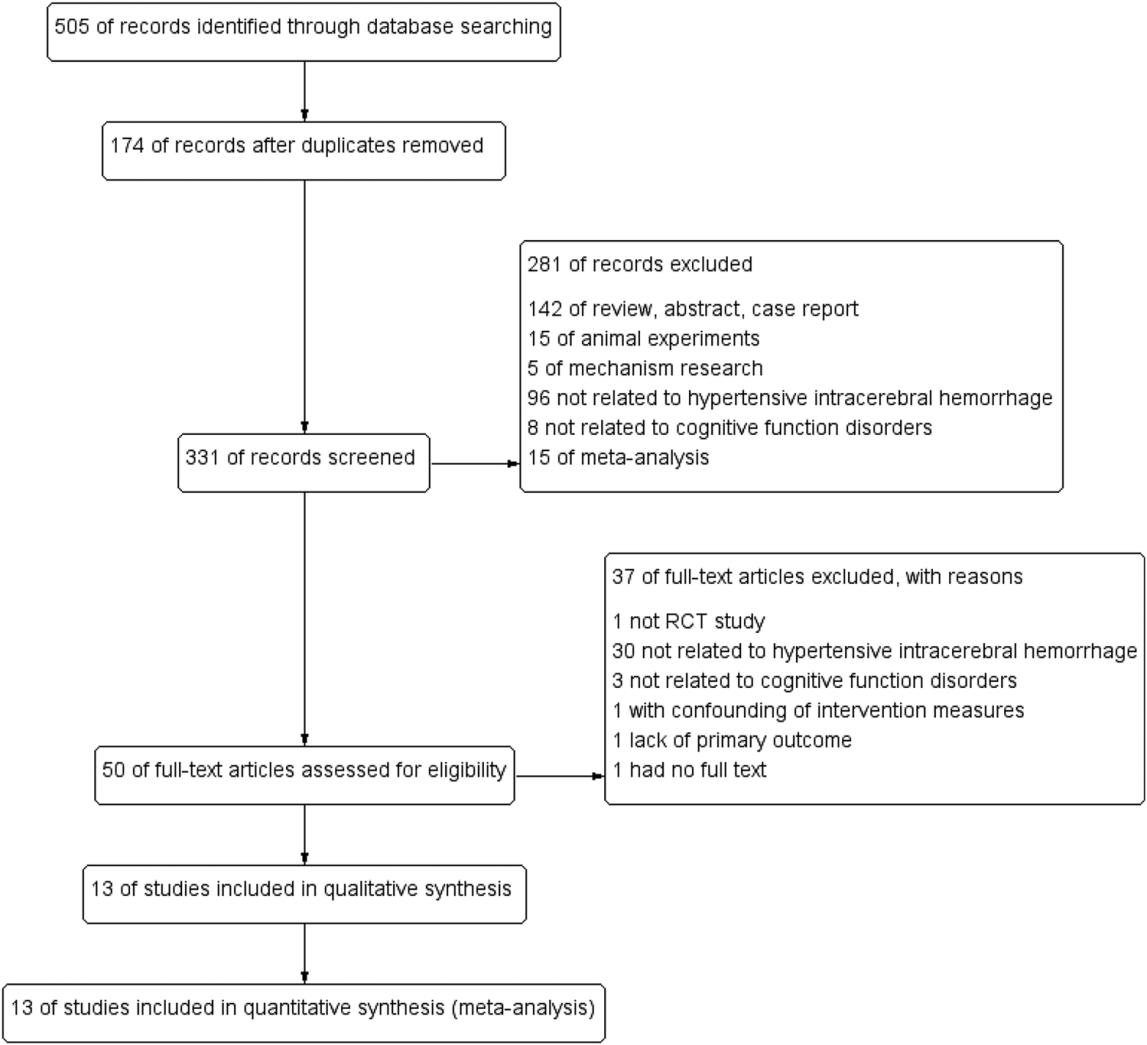
Flow chart for study selection.

A total of 505 studies were retrieved from all databases. After excluding 174 duplicate entries, 281 studies were eliminated during the title and abstract screening phase. A full‐text review was conducted on 50 selected studies. Subsequently, 37 studies were excluded for various reasons: one study was not a RCT, 30 were unrelated to HICH, three lacked relevance to cognitive function disorders, one involved confounding intervention measures, one did not report a primary outcome, and one was missing a complete text. Finally, 13 studies (Ao and Hu 2021; Cai 2022; J. Li and Fu 2020; Luo et al. 2013; Ni, Lv, Xu, and Peng 2023; Shi 2023; Tang et al. 2019; Tao et al. 2019; Yan and Liu 2024; H. Zhang and Chen 2023; B. Zheng 2022; L. Zheng 2023; J, Liu 2021) were included in the quality evaluation, encompassing a total of 561 cases in the ITCWM group and 562 cases in the control group (Figure 1).

### 3.2 Characteristics of Included Literature

All 13 studies were RCTs. Among these, four studies utilized a singular TCM therapy, while the remaining nine studies investigated the effects of multiple TCM therapies within the ITCWM group (Table 1). The outcomes assessed encompassed various measures: NIHSS score (*n *= 6), modified Barthel index (MBI, *n *= 2), ADL score (*n *= 2), Mini‐Mental State Examination (MMSE) score (*n *= 7), Loewenstein Occupational Therapy Cognitive Assessment (LOTCA) score (*n *= 3), Montreal Cognitive Assessment (MoCA) score (*n *= 2), TCMS scores (*n *= 5), total effective rate (*n *= 9), and adverse events rate (*n *= 5).

**TABLE 1:** Characteristics of eligible studies.

First author	Year	ITCWM group				Control group				Therapy duration	Outcomes	Total effective rate definition
		Size	Age (mean ± SD)	Gender (M/F)	Interventions	Size	Age (mean ± SD)	Gender (M/F)	Interventions			
Shi (2023)	2023	40	64.36 ± 1.42	22/18	traditional Chinese medicine decoction + scalp acupuncture therapy + conventional treatment	40	64.27 ± 1.38	23/17	Conventional treatment	—	NIHSS score, QOL, LOTCA score, TCMS score, adverse events	—
Zheng (2022)	2022	46	66.2 ± 10.3	23/23	traditional Chinese medicine + scalp acupuncture + conventional treatment	46	65 ± 10.8	26/20	Conventional treatment	8 weeks	LOTCA score, TCMS score, total effective rate, adverse events	Basic recovery (NIHSS reduction ≥ 90%), significant improvement (46%–89%), effective (18%–45%), ineffective (< 18%).
H. Zhang and Chen (2023)	2023	40	64.49 ± 10.19	20/20	acupuncture‐moxibustion and acupoint massage + hyperbaric oxygen therapy	40	63.59 ± 10.24	25/15	Hyperbaric oxygen therapy	2 weeks	NIHSS score, ADL, MoCA score, total effective rate	Significant improvement (consciousness recovery, normal thinking/reaction), improvement (significant consciousness improvement), ineffective (no relief).
Li and Fu (2020)	2020	30	47.12 ± 10.64	17/13	Buyang Huanwu decoction + conventional treatment	30	47.77 ± 10.22	20/10	Conventional treatment	14 days	MMSE score, QOL, ADL, total effective rate	Recovery (normal ADL, significant hematoma absorption), improvement (partial recovery), ineffective (no self‐care ability).
Jing Liu [2021]	2021	49	55.57 ± 4.49	24/25	Huoxue Ditan decoction + Buyang Huanwu decoction + conventional treatment	49	55.46 ± 4.38	25/24	Conventional treatment	21 days	NIHSS score, MMSE score, total effective rate, adverse events rate	Basic recovery (NIHSS reduction ≥ 90%), improvement (NIHSS reduction ≥ 50%), ineffective (< 50%).
Tang et al. (2019)	2019	31	58.32 ± 4.56	17/14	traditional Chinese medicine decoction + conventional treatment	31	58.21 ± 4.67	16/15	Conventional treatment	3 weeks	NIHSS score, MMSE score, adverse events rate	—
Cai (2022)	2022	25	63.45 ± 8.38	13/12	traditional Chinese medicine decoction + electroacupuncture therapy + conventional treatment	25	63.51 ± 8.26	14/11	Conventional treatment	8 weeks	TCMS score, total effective rate	Ineffective (improvement MoCA score < 20% or worsening), effective (improvement > 50%), significant improvement (improvement > 85%)
Tao et al. (2019)	2019	45	59.86 + 7.09	25/20	acupuncture + modified Lingjiao Gouteng decoction + conventional treatment	45	61.11 + 7.18	28/17	Conventional treatment	2 weeks	NIHSS score, MMSE score, total effective rate	Significant improvement (consciousness recovery, normal thinking/reaction), improvement (significant consciousness improvement), ineffective (no relief).
L. Zheng (2023)	2023	75	50.15 ± 3.24	53/22	traditional Chinese medicine + scalp acupuncture + conventional treatment	75	49.56 ± 3.19	47/28	Conventional treatment	2 weeks	LOTCA score, TCMS score, total effective rate, adverse events rate	Basic recovery (NIHSS reduction ≥ 90%), improvement (46%–89%), ineffective (< 18%).
Ni et al. (2023)	2023	32	57 ± 10	24/8	Electroacupuncture treatment + conventional treatment	34	60 ± 8	24/10	Conventional treatment	2 weeks	NIHSS score, Barthel index, MMSE score, total effective rate	NIHSS score reduction rate ≥ 46% (effective), 18%–46% (improved), < 18% (ineffective).
Luo et al. (2013)	2013	54	/	—	traditional Chinese medicine decoction + acupuncture therapy + conventional treatment	53	—	—	Conventional treatment	22 days	MMSE score	—
Ao and Hu (2021)	2021	41	62.1 ± 10.4	26/15	traditional Chinese medicine + scalp acupuncture therapy + conventional treatment	41	62.3 ± 10.8	25/16	Conventional treatment	8 weeks	TCMS score, total effective rate	Basic recovery (NIHSS reduction ≥ 90%), significant improvement (46%–89%), effective (18%–45%), ineffective (< 18%).
Yan and Liu (2024)	2024	53	72.78 ± 4.34	29/24	traditional Chinese medicine + acupuncture + acupoint massage + conventional treatment	53	73.02 ± 4.60	30/23	Conventional treatment	8 weeks	Barthel index, MMSE score, MoCA score	—

Abbreviations: ADL, activity of daily living score; Barthel, Modified Barthel Index; ITCWM, integrated traditional Chinese and Western medicine; LOTCA, Loewenstein Occupational Therapy Cognitive Assessment; MMSE, Mini‐Mental State Examination; MoCA, Montreal Cognitive Assessment; NIHSS, National Institute of Health Stroke Scale; TCMS, traditional Chinese medicine (TCM) syndrome integral scale.

### 3.3 Quality Assessment

**FIGURE 2:**
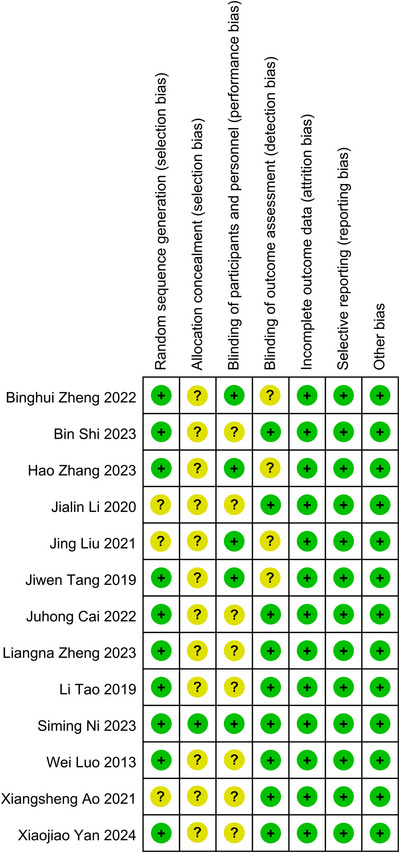
Quality assessment was analyzed by the Cochrane risk bias tool.

The quality of the enrolled studies was assessed utilizing the Cochrane tool, which encompasses three dimensions: low risk, high risk, and uncertain risk of bias. The findings indicated that all studies demonstrated high to moderate quality (Figure 2). Consequently, all 13 studies were included in the meta‐analysis.

### 3.4 NIHSS Score

**FIGURE 3:**
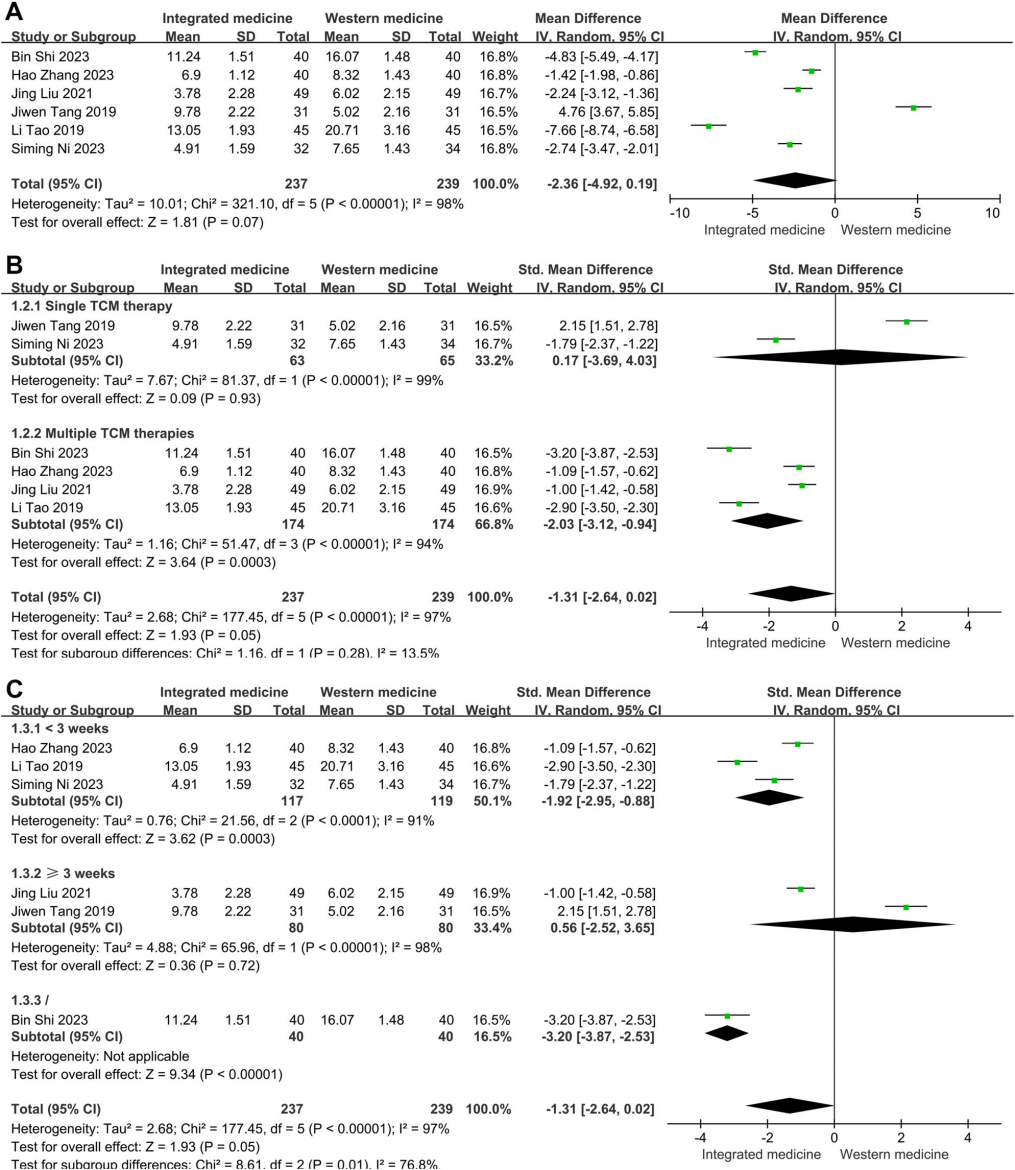
Effects of ITCWM on NIHSS scores in patients exhibiting post‐HICH cognitive dysfunction. (A) Pooled results. (B) Subgroup analysis for NIHSS score based on interventions implemented within the ITCWM group. (C) Subgroup analysis for NIHSS score based on therapy duration.

A marked heterogeneity was identified in the pooled analysis concerning the NIHSS score (*p* < 0.001, *I* ^2^ = 98%). Then, these indexes were analyzed based on the random‐effect model. Summary results revealed a significant effect of ITCWM in patients with post‐HICH cognitive dysfunction. The overall analysis demonstrated that the NIHSS score (SMD = 2.36, 95% CI: −4.92, 0.19, Figure 3A) was significantly lower in the ITCWM cohort compared to the Western medicine group. It suggested that ITCWM could significantly improve the neurological deficits of patients.

Subgroup analysis was conducted to explore the sources of heterogeneity based on therapy duration (< 3 weeks vs. ≥ 3 weeks), the type of TCM intervention (single TCM therapy vs. multiple TCM therapies). Nevertheless, heterogeneity persisted across these subgroups. Subgroup analysis indicated that patients receiving multiple TCM therapies exhibited a significant reduction in NIHSS scores (SMD = −2.03, 95% CI: −3.12, −0.94, Figure 3B). In addition, when stratified by therapy duration, patients treated for less than 3 weeks also demonstrated a significant reduction in NIHSS scores (SMD = −1.92, 95% CI: −2.95, −0.88, Figure 3C).

### 3.5 ADL Scores

**FIGURE 4:**
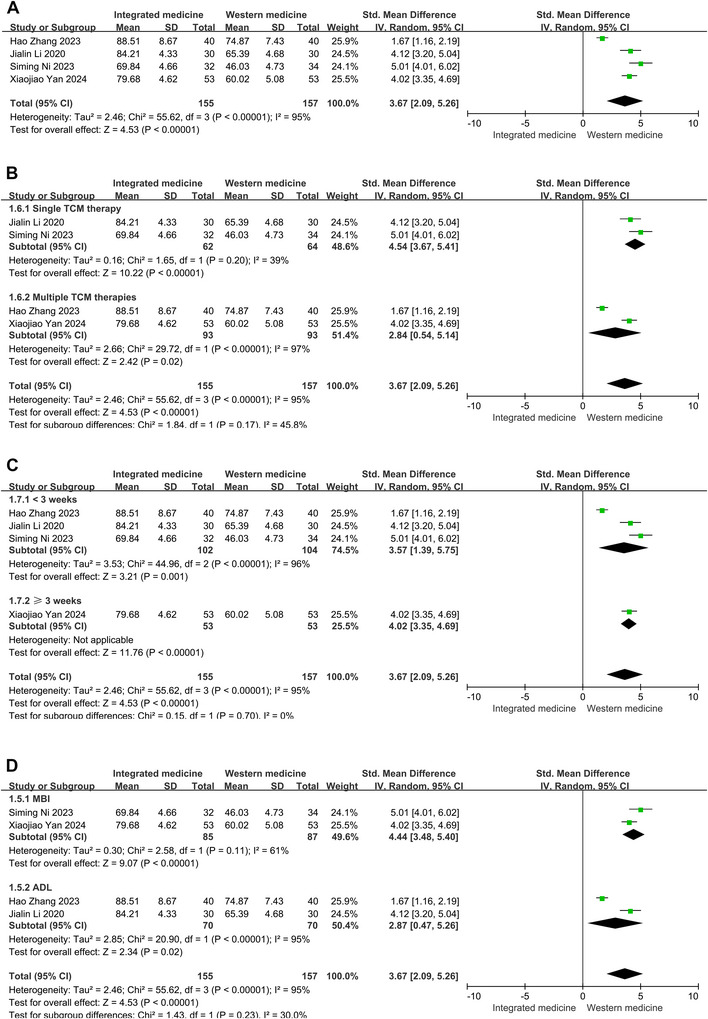
Effects of ITCWM on the ADL scores of patients experiencing post‐HICH cognitive dysfunction. (A) Pooled results. (B) Subgroup analysis for ADL scores based on interventions of the ITCWM group. (C) Subgroup analysis for ADL scores based on therapy duration. (D) Subgroup analysis for ADL scores based on scale type.

A significant degree of heterogeneity was discovered in the pooled analysis for the ADL scores (*p* < 0.001, *I* ^2 ^= 95%). Consequently, these indexes were analyzed based on the random‐effect model. The results indicated that ITCWM significantly improved patients' ability to perform daily activities. Specifically, the analysis revealed that the ADL score was significantly higher in the ITCWM therapy group compared to controls (SMD = 3.67, 95% CI: 2.09, 5.26, Figure 4A).

Subgroup analysis was conducted to identify sources of heterogeneity based on therapy duration, the specific type of TCM intervention, and the assessment scales employed. The single TCM therapy group (*p* = 0.20, *I* ^2^ = 39%) and MBI group (*p* = 0.11, *I* ^2^ = 61%) had low heterogeneity. Subgroup analysis showed that both single TCM therapy (SMD = 4.54, 95% CI: 3.67, 5.41) and multiple TCM therapies (SMD = 2.84, 95% CI: 0.54, 5.14) resulted in significant enhancements in ADL scores (Figure 4B). Furthermore, patients receiving treatment for less than 3 weeks (SMD = 3.57, 95% CI: 1.39, 5.75) as well as those treated for greater than or equal to 3 weeks (SMD = 4.02, 95% CI: 3.35, 4.69) both exhibited marked improvements in ADL scores (Figure 4C). Notably, the ITCWM group showed significant enhancements in both the Barthel index (SMD = 4.44, 95% CI: 3.48, 5.40) and ADL scores (SMD = 2.87, 95% CI: 0.47, 5.26) when compared to the control group (Figure 4D).

### 3.6 Cognitive Function

**FIGURE 5:**
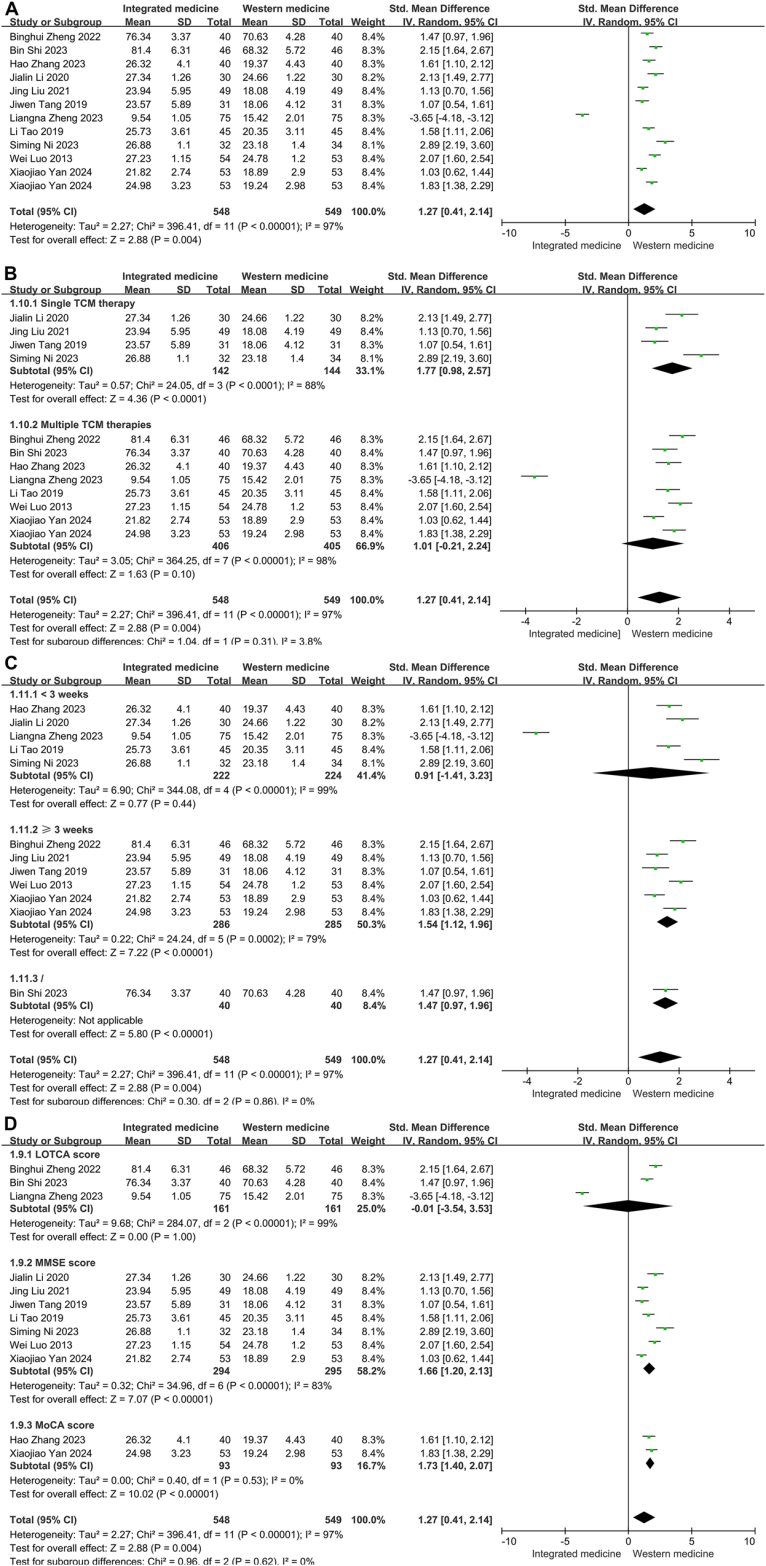
Effects of ITCWM on the cognitive function of patients experiencing post‐HICH cognitive dysfunction. (A) Pooled results. (B) Subgroup analysis for cognitive function based on interventions of ITCWM group. (C) Subgroup analysis for cognitive function based on therapy duration. (D) Subgroup analysis for cognitive function based on scale type.

High heterogeneity was discovered in the pooled analysis for cognitive function (*p* < 0.001, *I* ^2^ = 97%). Then, these indexes were analyzed based on the random‐effect model. ITCWM demonstrated a marked enhancement in cognitive function based on 12 enrolled tests. The overall analysis showed that the cognitive function score was elevated in the ITCWM group (SMD = 1.27, 95% CI: 0.41, 2.14, Figure 5A).

Subgroup analyses were performed to investigate the sources of heterogeneity based on therapy duration, the type of TCM intervention, and the measurement scales employed. The findings indicated that each of these factors, therapy duration, TCM intervention type, and the scale types, contributed to the observed heterogeneity (Figure 5). Subgroup analysis revealed that single TCM therapy (SMD = 1.77, 95% CI: 0.98, 2.57) had a more pronounced effect than multiple TCM therapies (SMD = 1.01, 95% CI: −0.21, 2.24) (Figure 5B). Notably, patients who underwent treatment for greater than or equal to 3 weeks demonstrated significant improvements in cognitive function (SMD = 1.54, 95% CI: 1.12, 1.96), whereas those receiving treatment for less than 3 weeks did not show any significant difference (SMD = 0.91, 95% CI: −1.41, 3.23) (Figure 5C). Analysis across different scales indicated that both MMSE (SMD = 1.66, 95% CI: 1.20, 2.13) and MoCA (SMD = 1.73, 95% CI: 1.40, 2.07) scales demonstrated significant effects of ITCWM (Figure 5D).

### 3.7 TCMS Score

**FIGURE 6:**
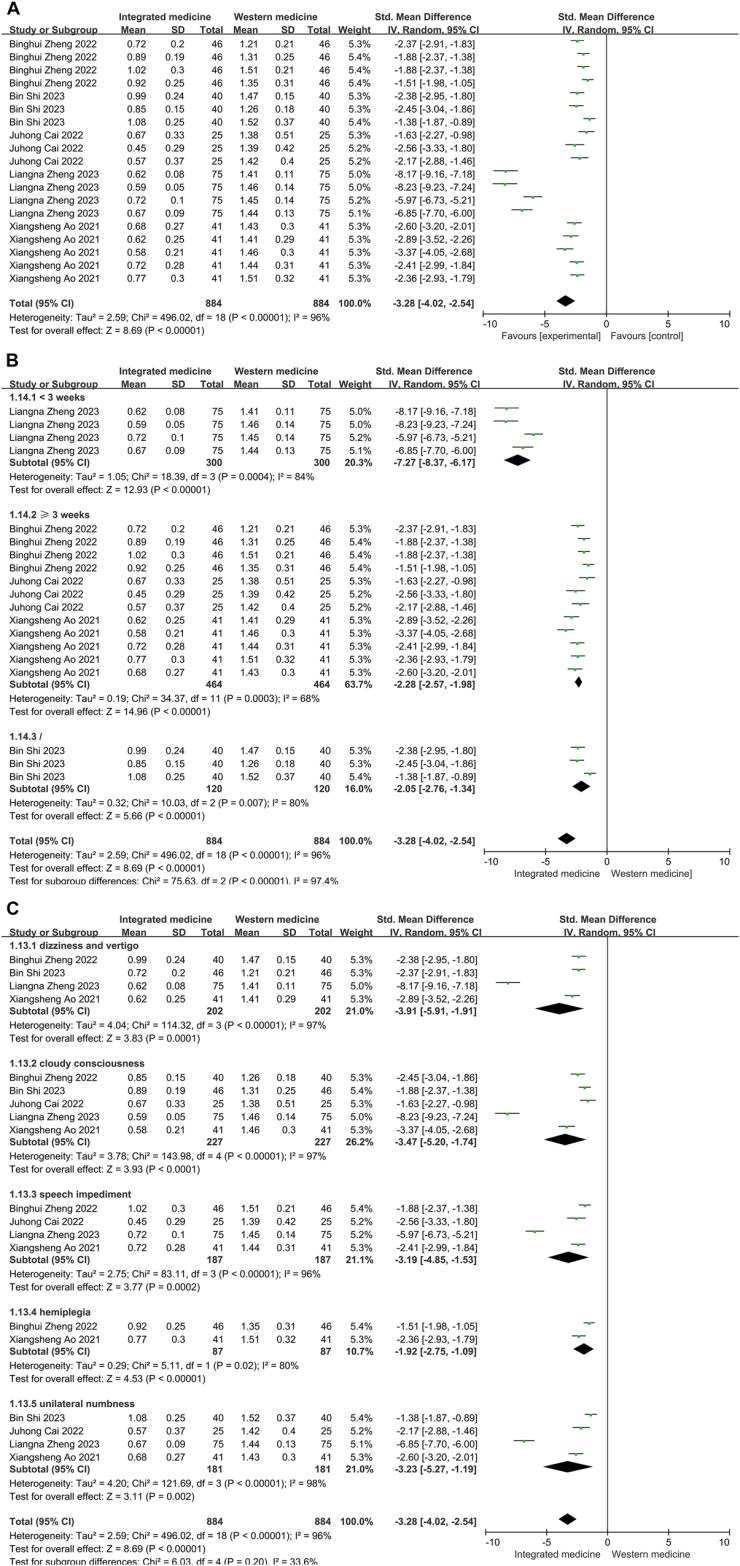
Effects of ITCWM on the TCMS score of patients experiencing post‐HICH cognitive dysfunction. (A) Pooled results. (B) Subgroup analysis for TCMS score based on therapy duration. (C) Subgroup analysis for TCMS score based on domain.

High heterogeneity was discovered in the pooled analysis for the activity of the TCMS score (*p* < 0.001, *I* ^2^ = 96%). Then, these indexes were analyzed based on the random‐effect model among 19 tests. The intervention of ITCWM notably led to a reduction in the TCMS score. The pooled analysis clearly indicated a substantial decrease in the TCMS score within the ITCWM group (SMD = −3.28, 95% CI: −4.02, −2.54, Figure 6A).

Subgroup analysis by therapy duration indicated that both patients receiving treatment for less than 3 weeks (SMD = −7.27, 95% CI: −8.37, −6.17) and those undergoing therapy for greater than or equal to 3 weeks (SMD = −2.28, 95% CI: −2.57, −1.98) showed significant reductions in syndrome scores (Figure 6B). Analysis across various domains showed substantial decreases in scores related to dizziness and vertigo (SMD = −3.91, 95% CI: −5.91, −1.91), cloudy consciousness (SMD = −3.47, 95% CI: −5.20, −1.74), speech impediment (SMD = −3.19, 95% CI: −4.85, −1.53), hemiplegia (SMD = −1.92, 95% CI: −2.75, −1.09), and unilateral numbness (SMD = −3.23, 95% CI: −5.27, −1.19) were all significantly decreased (Figure 6C).

### 3.8 Total Effective Rate and Adverse Events

**FIGURE 7:**
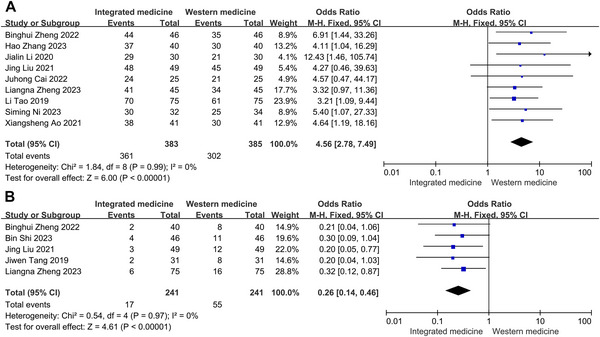
Effects of ITCWM on the total effective rate and adverse events in patients with post‐HICH cognitive dysfunction. (A) Pooled results for total effective rate. (B) Pooled results for adverse events.

Nine studies were conducted to analyze the overall effective rate, while five studies focused on adverse events. Remarkably, no significant heterogeneity was discovered in the pooled analyses for total effective rate (*p* = 0.99, *I* ^2^ = 0%) and adverse events (*p* = 0.97, *I* ^2^ = 0%). Consequently, the analyses were carried out using a fixed‐effect model. The impact of ITCWM on the total effective rate did not reach statistical significance. However, the summary results indicated that total effective rate was higher in ITCWM group (RR = 4.56, 95% CI: 2.78, 7.49, Figure 7A). Furthermore, the pooled data indicated a marked reduction in adverse events within the ITCWM group (RR = 0.26, 95% CI: 0.14, 0.46, Figure 7B).

### 3.9 Sensitivity Analysis and Publication Bias

**FIGURE 8:**
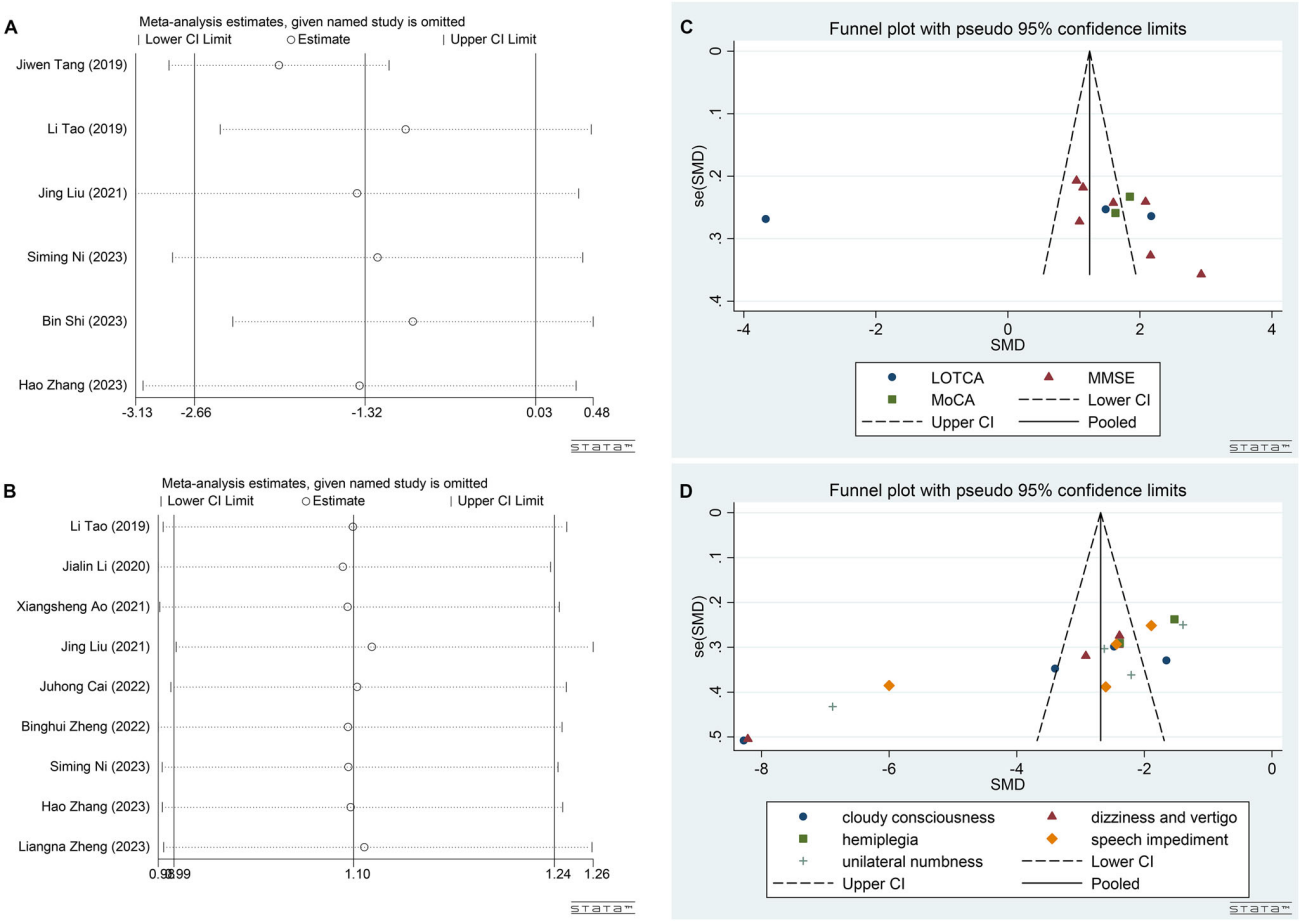
Sensitivity analysis and publication bias. (A) Sensitivity analysis for NIHSS score. (B) Sensitivity analysis focusing on the total effective rate. (C) Examination of publication bias related to cognitive function. (D) Publication bias concerning the traditional Chinese medicine syndrome score.

Sensitivity analysis revealed that the results from the combined analysis exhibited remarkable stability (Figure 8A,B). Furthermore, the assessment of publication bias indicated no substantial evidence of bias within the included studies (*p *> 0.05, Figure 8C,D).

## 4 Discussion

This meta‐analysis meticulously assessed the efficacy and safety of ITCWM in managing cognitive dysfunction following HICH. The results demonstrated that ITCWM significantly improved neurological deficits, activities of daily living, cognitive function, TCMS scores, and total effective rate, while reducing the incidence of adverse events. These results provide a solid foundation for further exploration of the underlying mechanisms, clinical applications, and prospective research avenues pertaining to ITCWM in the management of cognitive dysfunction post‐HICH.

The meta‐analysis yielded several significant findings. First, the application of ITCWM resulted in a notable reduction in NIHSS scores, indicative of enhanced neurological function. This finding corroborates previous research that highlights the efficacy of TCM therapies, which, by promoting blood circulation and resolving stasis, can effectively diminish brain edema and facilitate hematoma absorption (J. Zhou, Sun et al. 2024). Second, a substantial improvement in ADL scores was observed with ITCWM. This may be ascribed to the synergistic effects of TCM in conjunction with Western medicine, which collectively enhance motor function and cognitive capabilities through multi‐target mechanisms (L. Li et al. 2024; J. Zhou et al. 2022). Third, cognitive function scores were significantly elevated in the ITCWM cohort, particularly among patients receiving treatment for greater than or equal to 3 weeks. This suggests that extended treatment durations may be necessary to attain optimal cognitive improvements. Fourth, there was a significant reduction in TCMS scores, reflecting the efficacy of ITCWM in alleviating specific symptoms associated with TCMS, such as dizziness, vertigo, and speech impediment. Finally, the association of ITCWM with a decreased incidence of adverse events further underscores its safety profile. These results collectively indicate that ITCWM therapy is both effective and safer than conventional Western medical approaches in the management of post‐HICH cognitive dysfunction. This aligns with prior findings from a meta‐analysis conducted by Zhen et al., which reported that Buyang Huanwu decoction demonstrated a superior efficacy rate and a lower incidence of adverse events compared to antidepressant medications (Zhen et al. 2022).

The notable reduction in NIHSS scores can be explained by the synergistic effects of TCM and Western medicine. TCM prescriptions regulate inflammatory responses and improve microcirculation, providing a better physiological basis for acupuncture treatment. TCM therapies, such as Buyang Huanwu decoction, glycosides modulated the foam cells, lipid metabolism, inflammation factors, and adhesion molecules of macrophages in atherosclerosis via the JAK/STAT pathway (Fu et al. 2022), while Western medicine interventions like minimally invasive hematoma evacuation address structural damage. They are particularly effective in the early stages of recovery, where reducing brain edema and promoting hematoma absorption are critical. Acupuncture works by stimulating specific acupoints, which can enhance cerebral blood flow, reduce inflammation, and promote neurogenesis. Dong et al. found that acupuncture at the Baihui (GV20) and Qubin (GB7) points has been shown to reduce oxidative stress and neuronal apoptosis via the SIRT1/FOXO1 pathway in animal models of intracerebral hemorrhage (Dong et al. 2024). Acupuncture could regulate the autophagy via the JNK pathway in a rat model with intracerebral hemorrhage (Dai et al. 2023). It is particularly beneficial for improving attention and memory, as well as reducing symptoms of anxiety and depression that often accompany cognitive dysfunction (Y. Zhang, Tang et al. 2024). Each intervention has distinct mechanisms and indications, and their combined use can provide comprehensive benefits to patients. Future research should focus on further elucidating the specific pathways through which these interventions exert their effects and optimizing their application in clinical practice. The combination of TCM and acupuncture treatment shows more significant neural function recovery and brain tissue protection effects (Tao et al. 2019). Subgroup analysis revealed that patients undergoing multiple TCM therapies exhibited a significantly greater reduction in NIHSS scores when compared to those receiving a single TCM therapy. This suggests that a multifaceted TCM approach may enhance treatment efficacy. In addition, patients receiving treatment for less than 3 weeks demonstrated significant reductions in NIHSS scores, underscoring the potential that short‐term ITCWM can yield neurological improvements. Improvements in ADL scores may stem from the enhancement of motor and cognitive functions through postoperative rehabilitation, combined with the regulatory effects of TCM on cerebral microcirculation (X. Zhang, Pei et al. 2024). Notably, the profound impact of multiple TCM therapies on cognitive and motor functions. Both short‐term and long‐term treatments have been shown to significantly improve ADL scores, suggesting that ITCWM can augment daily living abilities regardless of treatment duration.

The observed enhancements in cognitive function, particularly with prolonged treatment durations, suggest that the neuroprotective effects of ITCWM require sustained intervention to manifest fully (Pei et al. 2024). A detailed subgroup analysis revealed that mono‐therapy using TCM exerted a more pronounced effect compared to combined TCM therapies, potentially due to the specific mechanisms of single herbs or formulations in addressing cognitive impairments. Notably, patients undergoing treatment for greater than or equal to 3 weeks exhibited significant cognitive function improvement, indicating that longer treatment durations are necessary to achieve optimal cognitive benefits. Different cognitive function scales (MMSE and MoCA) both showed significant positive effects of ITCWM, suggesting that the advantages of this treatment are consistent across various evaluative tools.

The reduction in TCMS scores reflects the holistic strategy employed by TCM in addressing symptom clusters, potentially linked to its effects on neuroinflammation and neuronal repair (Wu et al. 2024). Subgroup analysis stratified by therapy duration revealed that patients receiving treatment for less than 3 weeks, as well as those undergoing treatment for greater than or equal to 3 weeks, demonstrated significant decreases in syndrome scores. This finding suggests that ITCWM is capable of effectively alleviating symptoms associated with TCM syndromes, independent of the treatment duration. Furthermore, a detailed analysis across various domains indicated that scores for dizziness and vertigo, cloudy consciousness, speech impediment, and unilateral numbness were all significantly decreased, highlighting the comprehensive effectiveness of ITCWM in improving various symptoms. The reduced incidence of adverse events further emphasizes the safety of ITCWM, likely attributable to TCM's regulatory effects on the body's homeostasis (Liu et al. 2023). Subgroup analysis disclosed that both single TCM therapy and multiple TCM therapies were associated with diminished rates of adverse events, suggesting that ITCWM is safe regardless of the type of TCM intervention. In addition, patients undergoing treatment for greater than or equal to 3 weeks also exhibited a significant decline in adverse events, indicating that longer treatment durations do not compromise safety. The high stability of results and low publication bias further affirm the robustness of the present results.

Future research should be directed towards several directions. First, larger‐sample RCTs with standardized protocols are necessary to validate the efficacy and safety of ITCWM. Second, investigations should be undertaken to determine the optimal duration and dosage regimens of treatment in order to maximize therapeutic benefits. Third, mechanistic studies using advanced neuroimaging techniques and biomarker analyses have the potential to illuminate the specific pathways through which ITCWM exerts its effects. Fourth, long‐term follow‐up studies are crucial for evaluating the sustainability of cognitive improvements and functional outcomes. Finally, it is essential to explore the cost‐effectiveness of ITCWM in order to inform and guide healthcare policy decisions.

This meta‐analysis presents compelling evidence that ITCWM is both effective and safe in the management of post‐HICH cognitive dysfunction. The findings demonstrate that ITCWM significantly enhances neurological deficits, improves daily living abilities, boosts cognitive function, and increases the overall effective rate while reducing adverse events. Future research should prioritize the refinement of treatment protocols, elucidation of the underlying mechanisms, evaluation of long‐term outcomes, and establishment of a standardized ITCWM approach for addressing post‐HICH cognitive dysfunction.

## Author Contributions

 YX P and WQ P conceived and designed the experiments. YX P and WQ P performed the experiments. YX P and WQ P contributed sample collection and statistical analysis. All authors wrote the manuscript. All authors revised it critically for important intellectual content. All authors read and approved the final manuscript.

## Funding

The authors have nothing to report.

## Conflicts of Interest

The authors declare no conflicts of interest.

## Data Availability

The datasets used and/or analyzed during the current study are available from the corresponding author on reasonable request.
